# Associations between Traffic-Related Black Carbon Exposure and Attention in a Prospective Birth Cohort of Urban Children

**DOI:** 10.1289/ehp.1205940

**Published:** 2013-05-10

**Authors:** Yueh-Hsiu Mathilda Chiu, David C. Bellinger, Brent A. Coull, Shawn Anderson, Rachel Barber, Robert O. Wright, Rosalind J. Wright

**Affiliations:** 1 Department of Pediatrics, Icahn School of Medicine at Mount Sinai, New York, New York, USA; 2 Children’s Hospital Boston, Boston, Massachusetts, USA; 3 Department of Environmental Health, and; 4 Department of Biostatistics, Harvard School of Public Health, Boston, Massachusetts, USA; 5 Channing Laboratory, Brigham and Women’s Hospital & Harvard Medical School, Boston, Massachusetts, USA; 6 Department of Preventive Medicine, and; 7 Mindich Child Health and Development Institute, Icahn School of Medicine at Mount Sinai, New York, New York, USA

**Keywords:** attention, children, Conners’ Continuous Performance Test, hit reaction time, traffic-related air pollution, urban

## Abstract

Background: Ambient air pollution may have neurotoxic effects in children. Data examining associations between traffic-related air pollution and attention domains remain sparse.

Objectives: We examined associations between black carbon (BC), a marker of traffic particles, and attention measures ascertained at 7–14 years of age among 174 children in a birth cohort based in the Boston, Massachusetts, area.

Methods: We estimated BC levels using a validated spatial–temporal land-use regression model based on residence during children’s lifetime. Children completed the Conner’s Continuous Performance Test (CPT) measuring omission errors, commission errors, and hit reaction time (HRT), with higher scores indicating increased errors or slower reaction time. Multivariable-adjusted linear regression analyses were used to examine associations between BC and each attention outcome.

Results: Children were primarily Hispanic (56%) and Caucasian (41%); 53% were boys. We found a positive association between higher BC levels with increased commission errors and slower HRT, adjusting for child IQ, age, sex, blood lead level, maternal education, pre- and postnatal tobacco smoke exposure, and community-level social stress. Notably, the association was weaker, though still positive, for the highest BC quartile relative to the middle two quartiles. Sex-stratified analysis demonstrated statistically significant associations between BC and both commission errors and HRT in boys, but BC was not significantly associated with any of the CPT outcomes in girls.

Conclusions: In this population of urban children, we found associations between BC exposure and higher commission errors and slower reaction time. These associations were overall more apparent in boys than girls.

Evidence links exposure to traffic-related air pollution with a number of adverse health outcomes such as respiratory and cardiovascular disease, particularly in the urban environment (Bernstein 2012; Brunekreef et al. 2009; Fang et al. 2012; Iannuzzi et al. 2010). Although literature about potential neurocognitive effects is still sparse, increasing evidence suggests that chronic ambient air pollution exposure may have neurotoxic effects in children, with evidence implicating particulate matter (PM), with an aerodynamic diameter ≤ 2.5 µm (PM_2.5_) (Kannan et al. 2006). Particles may enter the body via the lungs through breathing or via the digestive system through ingestion, then translocate via the blood stream to other organs (Peters et al. 2006). Inhaled particles may be particularly toxic, because this entry route will bypass metabolic processing by the liver, allowing a proportionally larger dose of PM to reach the blood–brain barrier than particles that enter the blood via the digestive tract. Both animal and human studies show that inhaled particles may be translocated to the central nervous system (CNS). Oberdörster et al. (2004) found ultrafine elemental carbon-13 particles in the olfactory bulb, cerebrum, and cerebellum in rats after inhalation of particles. Elder et al. (2006) showed that ultrafine particles can reach the brain, either through circulation or by direct translocation to the olfactory bulb from the nose in children.

Studies also suggest that PM and other ambient pollutants may invoke a chronic inflammatory process in the respiratory tract resulting in systemic release of inflammatory and oxidative stress mediators (Campbell 2004). Such peripheral immune activation may lead to neuroinflammation when inflammatory signals in the periphery are communicated to the brain (Teeling and Perry 2009). Observational and experimental studies have reported evidence of neuroinflammatory changes in the CNS in association with chronic ambient air pollution exposure (Calderón-Garcidueñas et al. 2003, 2008b; Campbell et al. 2005). For example, Campbell et al. (2005) found that levels of proinflammatory cytokines [interleukin (IL)-1α and tumor necrosis factor (TNF)-α] and immune-related transcription factor [nuclear factor (NF)–κB] were elevated in brain of mice exposed to particulate matter (4 hr/day, 5 days/week, for 2 weeks), compared with control mice. Calderón-Garcidueñas et al. (2003) reported histological evidence of chronic brain inflammation among young canines exposed to air pollution (*n* = 26) compared with control group (*n* = 14). This group also examined neuropathology among autopsied healthy children and young adults who died suddenly; they found greater neuroinflammation among those who had lived in high-pollution areas (*n* = 35) compared with those from low-pollution areas (*n* = 12) (Calderón-Garcidueñas et al. 2008b). Another study documented magnetic resonance imaging (MRI) lesions and deficits of memory and executive function among children (Calderón-Garcidueñas et al. 2008a) living in highly polluted Mexico City (*n* = 55), compared with children from a less polluted area (*n* = 18).

Previous epidemiological studies have reported associations between ambient air pollution and deficits in global measures of cognitive functioning (IQ) and a number of specific domains including learning, memory, and executive function in children (Edwards et al. 2010; Guxens et al. 2011; Suglia et al. 2008a; Wang et al. 2009). These studies are important because understanding associations among air pollution exposure and a broad range of domain-specific neuropsychological tests may provide greater insight into the underlying CNS alterations that may be operating (e.g., structural changes or altered neural systems and neurotransmitter pathways) (White et al. 2009).

Attention problems are among the most common neurobehavioral conditions affecting youth. Inattention has implications for ongoing learning, social functioning, and academic achievement throughout the life course (Spira and Fischel 2005). Attention deficit symptoms, a specific executive function domain, may contribute to more serious conditions including conduct disorder and attention deficit/hyperactivity disorder (ADHD) (Spira and Fischel 2005). Although environmental factors have been implicated (e.g., exposure to prenatal tobacco smoke, alcohol, or lead, or perinatal stress) (Braun et al. 2006; Froehlich et al. 2009; Linnet et al. 2003), to our knowledge few studies have examined the association between ambient air pollution and attention deficit symptoms in children, including two cross-sectional studies (Siddique et al. 2010; Wang et al. 2009) and one prospective birth cohort (Perera et al. 2012).

The importance of understanding sex differences in the neurotoxicity of a number of environmental chemicals has been increasingly underscored (Vahter et al. 2007). Studies in the past decades have started to examine the role of sex differences when studying effects of environmental exposures on neurodevelopmental disorders, including cognitive function and learning disabilities (Cory-Slechta et al. 2008). Relatively few human studies have examined sex differences in toxicant-associated attention deficit symptoms in children. An analysis examining the association between environmental toxicants and ADHD using data from the National Health and Nutrition Examination Survey (*n* = 4,704) suggested that the association between prenatal maternal smoking and ADHD may be stronger in girls (Braun et al. 2006). On the other hand, a recent study of prenatal exposure to organochlorines and attention domains reported that the associations were observed only among boys (*n* = 258), not in girls (*n* = 254) (Sagiv et al. 2012). To our knowledge, sex differences in associations between ambient air pollution exposures and attention have not yet been examined.

In the present study, we addressed gaps in the research to date. We examined whether children’s increased lifetime exposure to black carbon (BC), a marker of traffic-related particles from gasoline- and especially diesel-powered motor vehicles, was associated with attention ascertained at 7–14 years of age in a birth cohort based in Boston, Massachusetts, that was followed prospectively. We also examined whether sex modified these associations.

## Materials and Methods

*Study participants*. These data were collected as part of a longitudinal pregnancy cohort study designed to evaluate the effects of pre- and postnatal tobacco smoke exposure on childhood lung growth and development and respiratory health as described elsewhere (Hanrahan et al. 1992). In March 1986–October 1992, 1,000 English- or Spanish-speaking pregnant women > 18 years old receiving prenatal care (< 20th week of gestation) at an urban community health center in Boston who planned to have pediatric follow-up at the clinic were recruited to the Maternal–Infant Smoking Study of East Boston (MISSEB); 848 of the mothers gave birth to a live-born infant and continued longitudinal follow-up.

Between November 1996 and December 1998, new study initiatives were implemented to assess social stressors and neurodevelopmental outcomes. Given budgetary constraints, neurocognitive assessments were completed on a randomly selected subset of 218 children covering a broad range of cognitive domains (Suglia et al. 2008a); 183 successfully completed the attention test, which was the last assessment in the 2.5-hr cognitive battery. There were no significant differences in sociodemographic characteristics, birth weight, blood lead level, or tobacco smoke exposure between those who completed the attention neurocognitive assessment and those who did not. Specifically, mothers were mostly ethnic minorities (55.3% and 57.6% Hispanic or African American in the larger cohort and subset, respectively) with a high school education or less (80.9% in the larger cohort and 79.7% in the subset), and about half of the children were female (50.5% in larger cohort and 52.5% in the subset). Estimated BC exposures were also similar between those in the larger cohort (median BC = 0.63 μg/m^3^; IQR = 0.55–0.70 μg/m^3^) and those who completed the attention assessment (median BC = 0.63 μg/m^3^; IQR = 0.53–0.69 μg/m^3^). All protocols were approved by the human studies committees at the Beth Israel Deaconess Medical Center and Brigham and Women’s Hospital. Voluntary informed maternal consent and child assent when age appropriate was obtained before participation in this portion of the study.

*Traffic-related air pollution*. We estimated children’s lifetime exposure to BC, based on residence over each child’s lifetime from birth up to the neurocognitive assessment (i.e., updated if they relocated). We used a previously validated spatiotemporal model for 24-hr measures of BC based on 6,021 observations from > 2,079 unique exposure days at 82 locations in greater Boston, as detailed elsewhere (Gryparis et al. 2007).

In brief, one-quarter of the monitoring sites were governmental or commercial facilities, and the rest were residential. Daily predictions were based on day-specific temporal factors, such as BC levels measured from a central monitor (representing overall area concentration on a particular day), meteorological, and other characteristics (e.g., weekday/weekend) of a particular day; spatial factors associated with a specific location, such as traffic activity (e.g., cumulative traffic density within 100 m based on geographic information systems, distance to nearest major roadway, population density, percentage of urbanization); and interaction terms between temporal meteorological predictors and the source-based geographic variables to account for space–time interactions. Spline regression methods were used to allow factors to nonlinearly predict exposure. Thin-plate splines (a two-dimensional extension of regression splines), a form of universal kriging extended to incorporate covariates for daily particle concentrations, were used to model longitude and latitude and capture additional spatial variability unaccounted for after including the deterministic spatial predictors in the model.

Models were fit separately for the warm (May–October) and the cold (November–April) seasons to allow for the effects of spatial, temporal, and spatial × temporal terms, as well as the smooth spatial and temporal trends, to vary by season. The coefficient of determination (*R*^2^) for the model over both seasons was 0.82. We calculated children’s BC exposure by averaging daily predictions over the lifetime of each child, and then categorized BC exposure into quartiles to account for potential nonlinearity. For children who moved within the period of interest, we used weighted averages of the exposure at both the old (for the days they stayed at this address) and the new address (for the days that they stayed at the new address), accounting for this change of address. Hence the overall exposure of these children took into account both addresses, and more specifically the days that the children lived at each address. The Spearman’s correlation between prenatal and postnatal BC levels was 0.96, so we were not able to include both of them in the analyses.

*Attention domains*. Children completed the Conners’ Continuous Performance Test (CPT) (Conners 2000), a task-based computerized assessment of attention disorders and neurological functioning, at 7–14 years of age. The CPT does not have any reading or literacy requirements and can be administered to children as young as 6 years of age. Basically, the test consists of letters flashing in succession and at variable rates on the computer screen. The child was instructed to push the space bar as quickly as possible in response to each letter except for “X.” Thus, this test assesses response inhibition (errors of commission) as well as vigilance (errors of omission). The primary outcomes of interest included omission errors (failing to respond to a target), commission errors (erroneously responding to a non-target), and hit reaction time (HRT; mean reaction time for all target responses).

All measures are expressed as standardized *t*-scores or percentiles, which represent the performance of the individual taking the test relative to the performance by children of the same age and sex in the standardization sample developed based on a normative database of clinical and nonclinical subjects (Conners 2000). Scores were generated by the Conners’ CPT software. Commission errors and HRT are expressed by *t*-scores. A *t*-score of 50 represents the average for the standardization sample with an SD of 10 (e.g., *t* = 40 indicates 1 SD below average). Omission errors were indexed by percentile, which represents the percentage of the standardization group who scored below an individual’s score (e.g., omission error percentile = 25 means that only 25% of the comparison group made fewer errors). Higher scores/percentiles indicate worse performance (e.g., more errors or slower reaction time). Higher scores in omission errors, commission errors, and overall HRT indicate inattentiveness. The CPT has been widely used, and prior studies have found that reaction times are particularly sensitive indicators of exposures to toxicants (White et al. 2009).

*Measurement of other risk factors for neurodevelopment*. Sociodemographics. Maternal age, race/ethnicity, and educational status were ascertained by questionnaires at enrollment; information about child’s date of birth and sex were ascertained by the first postnatal questionnaire.

Tobacco smoke exposure. At each clinic visit during pregnancy, mothers were asked about their smoking status and the smoking habits of household members. A creatinine-corrected urine cotinine level was also obtained (Hanrahan et al. 1992). Women were classified as never smoking during pregnancy if they always reported that they did not smoke and each urinary cotinine level was < 200 ng/mg creatinine; otherwise they were classified as smokers. Mothers also reported on children’s postnatal secondhand smoke exposure on the postnatal questionnaires (monthly through age 26 months, every 6 months between ages 26 months and 4 years, and annually thereafter). Postnatal secondhand smoke was categorized as a composite of early (birth to 25 months) or late (≥ 26 months) exposure given small sample size in the latter group; 54 children were exposed both early and late, and 13 had late exposure only. All children exposed to prenatal smoking were also exposed to secondhand smoke after birth.

Blood lead. Children in Massachusetts are mandated by law to have annual blood lead testing until 4 years, or until 6 years of age if they are considered to be at high risk (e.g., living in pre-1978 housing that is deteriorated or undergoing construction, having a sibling with lead poisoning). Blood lead testing was carried out at the certified state laboratory, and results were included in the child’s medical record. A physician blinded to the study aims obtained blood lead levels from medical records using a standardized extraction form. In the analysis, we used the “peak blood lead level” referring to the highest blood lead level recorded up to 6 years of age.

Child’s IQ. Child intelligence was assessed using the composite IQ score on the Kaufman Brief Intelligence Test (K-BIT) (Kaufman et al. 1990) at the time that the CPT was administered.

Community-level social stress. Community violence may be associated with children’s neurocognitive development (Cooley-Strickland et al. 2009), and it may co-vary with traffic-related pollution exposure (O’Neill et al. 2003). Children’s exposure to community violence was measured at 6.8 ± 1.6 (mean ± SD) years of age using the My Exposure to Violence questionnaire (Selner-O’Hagan et al. 1998; Thomson et al. 2002). Items reported included the frequency of hearing gunshots, witnessing and/or experiencing slapping/hitting/punching fights, knife attacks and/or shootings in their neighborhood, and the like. The multi-item survey was summarized into a continuous score with higher scores indicating greater exposure using Rasch modeling, as detailed previously (Suglia et al. 2008b). The exposure score was then categorized into low, medium, and high levels based on tertiles.

*Analysis.* Of the 183 children completing the CPT, 9 were excluded *a priori* because their BC levels (*n* = 5) or CPT measures (*n* = 4) were > 3 SDs from the mean values. Thus, 174 children were included in the final analyses. We used multivariable linear regression to examine associations between BC quartiles and each CPT outcome in separate models. Maternal race and educational status and child’s IQ, sex, age at CPT assessment, exposure to prenatal maternal smoking and postnatal secondhand smoke, log-transformed peak blood lead level, and community violence exposure were considered potential confounders. To ensure that the precision of the estimates was not affected by including the covariates listed above, we made sure that there were no signs of instability in the fitted model, such as unusually large estimates or standard errors. Effect modification by sex was examined in stratified analyses as well as by fitting interaction terms. Furthermore, we additionally fit a model that allowed the estimated effect of BC to vary by sex, but constrained the estimated effect of each additional confounder to be the same for males and females. This assumption of constant estimated effects of confounders across sex yields a more parsimonious model, potentially improving power to detect associations as long as the associations between other covariates and the outcome do not vary greatly by sex. All analyses were performed using SAS (version 9.1.3; SAS Institute Inc., Cary, NC).

## Results

Table 1 shows participant characteristics as well as the distributions of BC and CPT outcomes for all participants, and the distribution of covariates by BC quartiles. Overall, most mothers had ≤ 12 years of education (81%); children were primarily Hispanic (Hispanic ethnicity regardless of race; 56%) or Caucasian (non-Hispanic white; 42%), and 53% were girls. Ethnic minorities (Hispanic or non-Hispanic African American) were more likely to be exposed to higher levels of BC compared with Caucasians (Wilcoxon rank-sum test *p* < 0.01). Median commission error and HRT *t*-scores were both approximately 50, indicating that the performance of children in our study was similar to that of children in the normative group. On the other hand, the interquartile range of omission error percentile was 74–98 (median, 92), implying more omission errors compared with the average population.

**Table 1:** Participant characteristics by BC quartiles.

Characteristic	All participants (*n*=174)	BC quartiles^*a*^			
		1st(*n*=43)	2nd(*n*=44)	3rd(*n*=43)	4th (*n*=44)
Child’s sex [*n* (%)]
Female	93 (53. 4)	26 (60.5)	18 (40.9)	25 (58.1)	24 (54.6)
Male	81 (46.6)	17 (39.5)	26 (59.1)	18 (41.9)	20 (45.5)
Race/ethnicity [*n* (%)]
Caucasian^*b*^	72 (41.4)	31 (72.1)	18 (40.9)	9 (20.9)	14 (31.8)
Hispanic^*c*^	97 (55.8)	12 (27.9)	25 (56.8)	34 (79.1)	26 (59.1)
Other	5 (2.9)	0 (0.0)	1 (2.3)	0 (0.0)	4 (9.1)
Maternal education [*n* (%)]
>12 years	33 (19.0)	13 (30.2)	8 (18.2)	6 (14.0)	6 (13.6)
≤12 years	141 (81.0)	30 (69.8)	36 (81.8)	37 (86.0)	38 (86.4)
Community violence exposure [*n* (%)]
Low	79 (45.4)	25 (58.1)	17 (38.6)	13 (30.2)	24 (54.6)
Medium	38 (21.8)	7 (16.3)	11 (25.0)	12 (27.9)	8 (18.2)
High	57 (32.8)	11 (25.6)	16 (36.4)	18 (41.9)	12 (27.3)
Secondhand smoke exposure [*n* (%)]
Never exposed	113 (64.9)	20 (46.5)	31 (70.5)	33 (76.7)	29 (65.9)
Prenatal and postnatal^*d*^	44 (25.3)	14 (32.6)	10 (22.7)	7 (16.3)	13 (29.5)
Early postnatal only^*e*^	6 (3.4)	3 (7.0)	1 (2.3)	1 (2.3)	1 (2.3)
Late postnatal only^*f*^	11 (6.3)	6 (14.0)	2 (4.5)	2 (4.7)	1 (2.3)
BC level (µg/m^3^) [median (IQR)]	0.63 (0.54–0.69)	0.42 (0.36–0.47)	0.6 (0.59–0.61)	0.66 (0.64–0.67)	0.72 (0.7–0.82)
Peak BLL (µg/dL) [median (IQR)]	7 (5–11)	6 (5–10)	9 (6–11)	7 (5–8)	9 (5–13)
Age at CPT assessment (years) [median (IQR)]	10 (9–11)	10.3 (9.08–11.33)	10.1 (9.04–11.63)	9.5 (8.58–11.25)	10 (8.88–11.25)
Child’s IQ [median (IQR)]	96 (85–103)	102 (91–105)	97 (86–103)	88 (82–99)	93 (85–106)
Omission errors (percentile) [median (IQR)]	91.7 (74.3–98.1)	89.5 (74.3–98.1)	83.9 (70.2–97.3)	96.7 (80.6–98.3)	91 (69.1–98.1)
Commission errors (*t*-score) [median (IQR)]	51 (44.9–57.9)	50.4 (39.8–55.6)	51.9 (45.9–61)	51.1 (45.9–57.9)	50.1 (44.3–60.3)
HRT (*t*-score) [median (IQR)]	49.9 (40.5–60.0)	45.2 (40.3–57.5)	55.4 (42–62)	49.6 (43.7–61.7)	50.9 (36.9–58.6)
^***a***^Ranges of BC exposure for each quartile: 1st quartile, 0.28–0.53 μg/m3; 2nd quartile, 0.54–0.62 μg/m3; 3rd quartile, 0.63–0.69 μg/m3; 4th quartile, 0.70–0.99 μg/m3. ^***b***^Non-Hispanic white. ^***c***^Hispanic ethnicity regardless of race. ^***d***^Exposure to both prenatal maternal smoking and postnatal secondhand smoke. ^***e***^Exposure to secondhand smoke from birth to 25 months postnatally. ^***f***^Exposure to secondhand smoke postnatally at age ≥26 months.					

*Association between BC levels and attention parameters*. In the sample considered as a whole, BC exposure was associated with increased commission errors and slower HRT, adjusting for age, sex, maternal education, community violence, blood lead level, IQ, prenatal smoking and postnatal secondhand smoke exposure (Table 2). However, for both outcomes the positive associations were weaker for the highest BC quartile than for the middle two quartiles, and only the middle two quartiles were significantly different (*p* < 0.05) from the first quartile. Although the association between BC and omission errors was not statistically significant, we observed a similar pattern with positive effect estimates in the second and third quartile.

**Table 2:** Multivariable linear regression models examining BC quartiles in relation to attention measures in urban children at 7–14 years of age [β (95% CI)].

BC level (quartile)	Omission error(percentile)	Commission error(*t*-score)	Slower HRT(*t*-score)
1st	Reference	Reference	Reference
2nd	2.66 (–6.34 to 11.66)	6.15 (2.03 to 10.27)	6.51 (0.43 to 12.59)
3rd	4.89 (–4.71 to 14.49)	4.75 (0.36 to 9.14)	6.14 (–0.35 to 12.63)
4th	0.62 (–8.57 to 9.81)	3.32 (–0.87 to 7.51)	1.75 (–4.44 to 7.94)
Models adjusted for age, sex, maternal education, exposure to community violence, peak blood lead level, child’s IQ, prenatal maternal smoking, and postnatal secondhand smoke exposure (never exposed, exposed only to postnatal ­secondhand smoke, exposed to both prenatal maternal smoking and postnatal secondhand smoke).			

**Figure 1:**
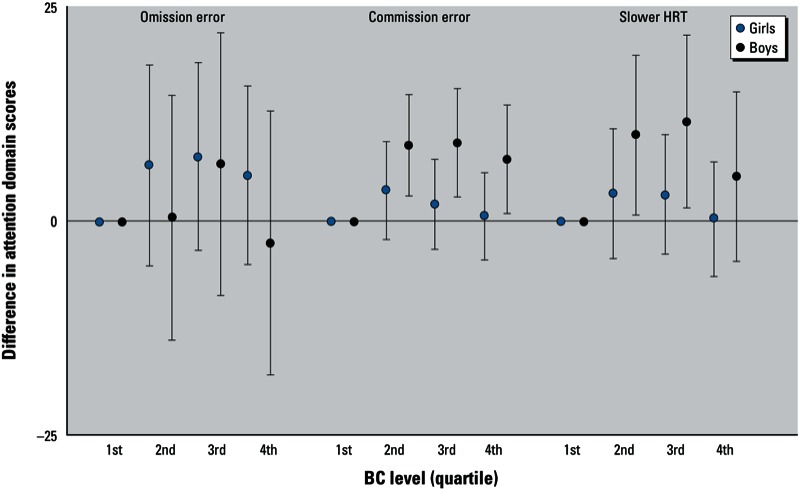
Sex-stratified associations between traffic-related BC exposure and attention domains in children 7–14 years of age. This figure demonstrates differences and 95% CIs in t-scores (for commission errors and HRT) or percentiles (for omission errors) across BC quartiles, stratified by sex. Higher scores or percentiles indicate more errors or slower HRT (indicators of inattentiveness). Models were adjusted for age at CPT assessment, maternal education, exposure to community violence, peak blood lead level, child’s IQ, prenatal maternal smoking, and postnatal secondhand smoke exposure (never exposed, exposed only to postnatal secondhand smoke, exposed to both prenatal maternal smoking and postnatal secondhand smoke).

*Effect modification by sex*. Figure 1 demonstrates the results from sex-stratified analyses. Among boys, associations with BC were significant for commission errors (β = 8.88, 95% CI: 2.64, 15.1 for BC 2nd quartile; β = 9.17, 95% CI: 1.54, 16.8 for 3rd quartile; and β = 7.25; 95% CI: –0.17, 14.7 for 4th quartile, compared with the lowest quartile). Similarly, BC was associated with slower HRT among boys (β = 10.1, 95% CI: 0.42, 19.8 for BC 2nd quartile; β = 11.7, 95% CI: –0.15, 23.6 for 3rd quartile; and β = 7.9, 95% CI: –6.24, 16.8 for 4th quartile, compared with the lowest quartile). In contrast, among girls associations of BC with commission errors and HRT were close to the null. Interaction *p*-values (BC × sex) were not significant at the α = 0.05 level (*p*_interaction_ with commission errors: BC 2nd quartile *p* = 0.27, 3rd quartile *p* = 0.20, 4th quartile *p* = 0.20; *p*_interaction_ with HRT: BC 2nd quartile *p* = 0.09, 3rd quartile *p* = 0.12, 4th quartile *p* = 0.10). The associations between BC quartiles and omission error percentiles were not significant for either sex, although they were more suggestive in girls. The analysis fitting a single multivariable-adjusted model that allowed the effect estimates of BC to vary by sex, but constrained the effect estimates of covariates to be the same for males and females, did not materially change these results.

## Discussion

To our knowledge, these are the first prospective data demonstrating an association between higher ambient BC exposure levels across childhood and attention outcomes in school-age urban children of lower socioeconomic status. Results suggest that boys may be more susceptible than girls to potential effects of traffic-related air pollution on some attention domains.

Our findings are generally similar to those of two previous cross-sectional studies comparing attention related outcomes based on children’s area of residence (i.e., polluted vs. clear, or urban vs. rural). Wang et al. (2009) compared neurobehavioral functions, including attention tests (e.g., continuous performance and visual simple reaction time), between students 8–10 years of age in Quanzhou, China, in a polluted area [*n* = 430; monthly average nitrogen dioxide (NO_2_) = 22 µg/m^3^] and those in a relatively clear area (*n* = 431; average NO_2_ = 7 µg/m^3^), and found that children in the polluted area in general had poorer performances after adjusting for age, body mass index, paternal education, sex, birth weight, delivery method, secondhand smoke, open kitchen, familiarity with computer games, vision, and breast feeding. Siddique et al. (2010) also found that the prevalence of ADHD, as screened following the criteria of the *Diagnostic and Statistical Manual of Mental Disorders* (DSM-IV) (American Psychiatric Association 1994), was significantly higher in Indian children residing in urban areas (*n* = 969; annual average NO_2_ = 50.1 µg/m^3^) than age- and sex-matched children from rural areas (*n* = 850; annual average NO_2_ = 30.3 µg/m^3^).

In our study, we assessed exposure using BC levels estimated based on place of residence over each child’s lifetime, which allowed us to quantify the traffic-related pollution levels and estimate associations between BC exposure and attention domains. Although no other study, to our knowledge, has estimated effects on attention related to an index of ambient traffic-related air pollution specifically, studies have considered other air pollutants such as polycyclic aromatic hydrocarbons (PAH), of which the major sources in urban areas are vehicular incomplete combustions and home heating. In a study of an urban birth cohort, Perera et al. (2012) found that prenatal PAH exposure measured by 48-hr personal monitoring was positively associated with a summed score of attention problems measured at 6–7 years of age using the Child Behavior Checklist (*n* = 253).

Although the difference in associations by sex was not statistically significant, the results from the stratified analyses suggest that boys may be more susceptible than girls to effects of traffic-related air pollution on attention, at least for some attention indicators. In boys, BC exposure above the first quartile was associated with more commission errors and slower hit reaction time than exposure in the lowest BC quartile, whereas in girls, associations with these outcomes were close to the null. To our knowledge, sex differences have not yet been examined comprehensively in the previous studies of air pollution and attention.

Notably, the association between BC and attention was overall weaker for the highest BC quartile of exposure than the middle two quartiles, suggesting a nonlinear relationship. This pattern is not readily explainable and warrants further study. It may be attributable to nonlinear concentration-dependent effects on underlying neurotransmitter systems (e.g., dopamine) (Monte-Silva et al. 2010) that may be operating in response to BC exposure. It is also possible that this pattern arises given differential distribution of co-varying factors across the BC exposure groups that contribute to the child’s underlying vulnerability to pollution effects. For example, our data showed that the children in the middle two BC quartiles also were exposed to higher levels of community violence (i.e., social stress) and had lower IQ than children in the highest quartile. Although we adjusted for these characteristics, there might have been confounding by other unmeasured factors that also varied according to exposure.

Evidence that social stress and physical environmental toxicants (e.g., air pollutants, chemicals) may influence common physiological pathways (e.g., oxidative stress, neruoendocrine and autonomic disruption, proinflammatory immune pathways) suggests potential additive and/or synergistic effects (Wright 2009). For example, we recently assessed cognitive function in 811 elderly men and found that the association between blood lead level and reduced cognition was stronger among those with higher perceived stress compared to those with lower perceived stress (*p*_interaction_ = 0.02) (Peters et al. 2010). Conversely, socially enriched environments may protect children from neurotoxicants (Laviola et al. 2008; Schneider et al. 2001). Previous findings suggest that children of higher IQ have a tendency to perform better on the CPT (Canfield et al. 2003). We had an insufficient number of study participants to evaluate modification of associations between BC and attention by child’s IQ or social stress. This should be examined in future research using larger samples and with more comprehensive measures of psychosocial stress.

In stratified analyses, the nonlinear pattern was seen in boys for commission errors and HRT and was suggested in girls for omission errors. The associations with BC quartiles were less precise for omission errors compared with other outcomes, possibly because of the relatively high rate of average omission errors and less stability for the percentile measurements in this study population; thus the results for omission errors need to be interpreted more cautiously. Also, we cannot rule out the possibility that the nonlinear pattern may have occurred due to chance.

Strengths of this study include the use of prospectively collected data, focus on lower SES and an ethnically mixed inner-city community sample, and the availability of data on many important confounders including lead exposure, tobacco smoke exposure, and child IQ. Other strengths include the assessment of traffic-related air pollution estimated using a validated spatiotemporal land-use regression model based on children’s residence during their lifetime, and a more subjective outcome assessment using a computerized task-based test that is not language dependent and unlikely to be affected by social norms such as questionnaire-based assessments of children’s attention.

In addition, we also acknowledge some limitations. Although we were able to adjust for several factors known to be important in children’s cognitive development, we did not have information on the child’s home environment. Although we had a measure of neighborhood-level stress (community violence), we did not have information on individual-level stressors that may influence neurocognitive function (Fishbein et al. 2009). We had low statistical power to estimate associations according to sex because the sample size was limited. Further studies in larger samples should consider sex-specific effects of traffic-related air pollution on attention in children. Further, although the CPT is a well-established tool that provides valuable information in clinical contexts and is appropriate for use in clinical assessments as part of a larger battery of tests, it is not meant to diagnose attention deficit disorders by itself. Finally, our study results may be more generalizable to children living in low-SES urban communities than other children.

Inattention and hyperactivity are highly and disproportionately prevalent among school-age urban youth, particularly among minorities, and have a negative impact on academic achievement (Basch 2011). Thus, understanding environmental risk factors that are amenable to intervention and prevention strategies is critical. To our knowledge, we are the first to report prospective data demonstrating an association between ambient BC exposure, an indicator of traffic-related particles, and attention in urban children. We found nonlinear associations of BC exposure with higher commission errors and slower reaction time, indicators of inattentiveness, in these urban school-aged children. In addition, these associations were more evident in boys than girls.
